# Novel Indole-Tethered Chromene Derivatives: Synthesis, Cytotoxic Properties, and Key Computational Insights

**DOI:** 10.3390/ph16030333

**Published:** 2023-02-22

**Authors:** M. Shaheer Malik, Hissana Ather, Shaik Mohammad Asif Ansari, Ayesha Siddiqua, Qazi Mohammad Sajid Jamal, Ali H. Alharbi, Munirah M. Al-Rooqi, Rabab S. Jassas, Essam M. Hussein, Ziad Moussa, Rami J. Obaid, Saleh A. Ahmed

**Affiliations:** 1 Department of Chemistry, Faculty of Applied Sciences, Umm Al-Qura University, Makkah 21955, Saudi Arabia; 2 Department of Pharmaceutical Chemistry, College of Pharmacy, King Khalid University (KKU), Abha 62529, Saudi Arabia; 3 Department of Clinical Pharmacy, College of Pharmacy, King Khalid University (KKU), Abha 62529, Saudi Arabia; 4 Department of Health Informatics, College of Public Health and Health Informatics, Qassim University, Al Bukayriyah 52741, Saudi Arabia; 5 Department of Chemistry, Jamoum University College, Umm Al-Qura University, Makkah 21955, Saudi Arabia; 6 Department of Chemistry, Faculty of Science, Assiut University, Assiut 71516, Egypt; 7 Department of Chemistry, College of Science, United Arab Emirates University, Al Ain 15551, United Arab Emirates

**Keywords:** chromenes, indole, multicomponent reaction, anti-cancer activity, molecular docking, ADME

## Abstract

Indole-tethered chromene derivatives were synthesised in a one-pot multicomponent reaction using *N*-alkyl-1*H*-indole-3-carbaldehydes, 5,5-dimethylcyclohexane-1,3-dione, and malononitrile, catalysed by DBU at 60–65 °C in a short reaction time. The benefits of the methodology include non-toxicity, an uncomplicated set-up procedure, a faster reaction time, and high yields. Moreover, the anticancer properties of the synthesised compounds were tested against selected cancer cell lines. The derivatives **4c** and **4d** displayed very good cytotoxic activity, with IC_50_ values ranging from 7.9 to 9.1 µM. Molecular docking revealed the potent derivatives have good binding affinity towards tubulin protein, better than the control, and the molecular dynamic simulations further demonstrated the stability of ligand-receptor interactions. Moreover, the derivatives followed all the drug-likeness filters.

## 1. Introduction

**Figure 1:**
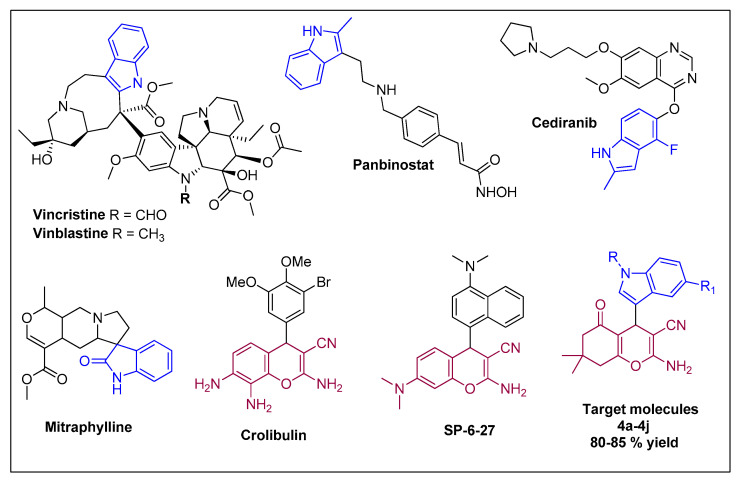
Anticancer agents with indole and chromene moieties.

Heterocyclic rings are fundamental structural components of many anticancer drugs. Almost three-quarters of the heterocyclic anticancer medications approved by the FDA between 2010 and 2015 are nitrogen-based heterocycles [1]. Their relevance in the design of anticancer drugs results from their ability to activate cell death and disrupt the biological processes associated with cancer growth [2,3]. Over the past several decades, indole and its derivatives have emerged as novel anticancer medicines that target many biological processes in cancer evolution [4]. Vincristine and vinblastine, the two most significant early indole-based anticancer medicines used to treat Hodgkin’s disease, are potent tubulin polymerization inhibitors with continued therapeutic relevance [5]. Recently, Novartis’ Panobinostat was licenced for treating multiple myeloma, and it is undergoing phase II studies to treat acute myeloid leukaemia [6]. Cediranib is another pan-VEGFR inhibitor that has demonstrated good preclinical efficacy in recurrent ovarian cancer [7]. Mitraphylline has also shown promising insights as a novel drug for treating both sarcoma and breast cancer in humans [8]. The antiproliferative and cytotoxic properties of mitraphylline were tested on MHH-ES-1, Ewing’s sarcoma, and MT-3 cell lines of breast cancer, and it suppressed the development of both cell lines at micromolar concentrations [9] (Figure 1).

Chromenes, specifically 4*H*-chromene and its derivatives, are found in different natural products, and they have a wide array of biological properties, comprising anti-microbial, anti-viral, anti-inflammatory, anti-tumor, anti-oxidant, anti-Alzheimer’s, anti-Parkinson’s disease, anti-HIV1, and anticonvulsant properties [10,11]. Due to its diverse biological effects, several synthetic derivatives of chromenes have been reported, some of which are used as potent medications and others are undergoing clinical trials [12,13]. Crolibulin (EPC2407), a synthetic chromene derivative, is undergoing a phase II clinical trial at the National Carcinoma Institute (NCI) for anaplastic thyroid cancer [14], and SP-6-27 has been selected for in vivo testing [15] (Figure 1).

As discussed above, natural and synthetic compounds with indole and chromene scaffolds are cytotoxic. Consequently, indole-tethered chromene derivatives may likewise exhibit diverse biological activities. Our research interest [16,17] and the importance of these scaffolds provide the impetus for the design and synthesis of new indole-tethered chromene derivatives through a multicomponent reaction catalysed by DBU, followed by the assessment of their anticancer capabilities against targeted cancer cells. Different computational investigations, including in silico and molecular dynamics studies, ADME, and drug-likeness calculations, were also conducted to determine the potential of the novel compounds. These synthesised compounds display pharmacophoric similarities and notable cytotoxic properties.

## 2. Results and Discussion

### 2.1. Chemistry

**Scheme 1:**
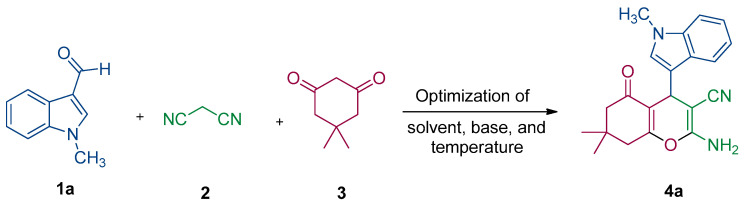
Model reaction to obtain product **4a**.

Using the appropriate substrates, a one-pot multicomponent reaction was used to generate the desired indole-tethered chromene derivatives. To optimise the reaction conditions, a model reaction was performed by using *N*-methyl-1*H*-indole-3-carbaldehyde **1a**, 5,5-dimethylcyclohexane-1,3-dione **2** and malononitrile **3** to synthesise **4a** (Scheme 1). Various reaction parameters, including temperature, solvent, and base, were evaluated to determine the ideal reaction conditions. For the optimization study, 1,8-dazabicyclo [5.4.0]undec-7-ene (DBU, a non-nucleophilic base) and pyridine (a weak nucleophilic base) bases were used as catalysts in protic (ethanol and methanol) and aprotic (acetonitrile and DMF) solvents at 60–65 °C. Delightfully, the desired reaction proceeded both in DBU and pyridine in protic and aprotic solvents at 60–65 °C in 1–3 h of reaction time, affording the product in varied yields. However, lower reaction yields with longer reaction times were observed when using pyridine as a base in both protic and aprotic solvents when compared to DBU. The reaction conducted with DBU in both protic and aprotic solvents showed a significant decrease in reaction time and an improvement in reaction yield. However, the reaction with DBU in aprotic solvents showed a lower yield (ca. 75% yield) and an extended reaction time (120 min) than in protic solvents. Compared to methanol, ethanol delivered the best yield of 85% in a short reaction time of 60 min for protic solvents. The optimization of the temperature demonstrated that 60–65 °C was better with respect to reaction time and yield, while 70–75 °C showed no significant enhancement (Table 1). With DBU in ethanol at 60–65 °C as optimised conditions, further optimization was performed by varying the quantity of DBU (Table 2). The results indicated that an optimum yield of **4a** with the shortest reaction time (60 min) was observed when 0.3 equivalent of DBU was used in ethanol at 60–65 °C.

**Scheme 2:**
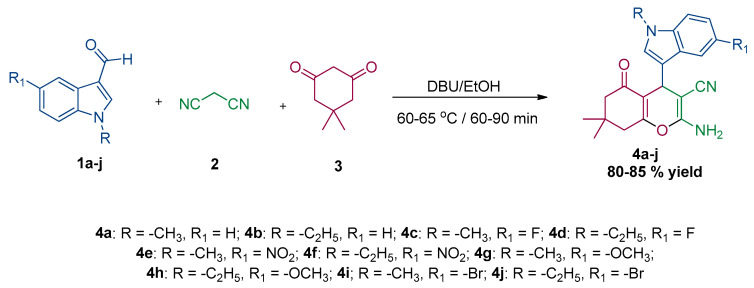
Synthesis of novel indole tethered chromene derivatives **4a**–**j**.

The standardised reaction condition was then extended to a variety of *N*-substituted indole-3-carbaldehydes **1a**–**j** containing electron-releasing (OMe) and electron-withdrawing groups (F, Br, and NO_2_) at the 5th position of the indole ring, malononitrile **2**, and 5,5-dimethylcyclohexane-1,3-dione **3** (Scheme 2). The newly developed one-pot, three-component reaction was equally efficient with different substituted indole-3-carbaldehydes, producing the derivatives **4a**–**j** in good yields of 80–85%. The products **4a**–**j** were completely characterised by nuclear magnetic resonance, infrared, and mass spectroscopy.

### 2.2. Cytotoxicity Studies

The novel indole-tethered chromene derivatives **4a**–**j** were assessed for their anticarcinoma properties against the three different human cancer cell lines, A549 (lung carcinoma), PC-3 (prostate carcinoma), and MCF-7 (breast carcinoma). Doxorubicin was used as a standard reference drug, and the results showed that most of the derivatives possess good cytotoxic properties. Among all compounds, **4c** and **4d,** having a fluorine substituent at the 5th position in the indole ring, were found to be the most potent derivatives against all the tested cell lines, with IC_50_ values ranging from 7.9 to 9.1 µM. In addition, compounds **4g** and **4h** showed moderate inhibitory activity with IC_50_ values of 10.5–12.6 µM against the three cell lines. Further, compounds **4e** and **4f** were less potent in MCF-7, with IC_50_ values between 58.9 and 62.8 µM, and they did not show any inhibitory activity against A549 and PC-3 cells. The inhibitory activity was also low for compounds **4a**, **4b**, **4i,** and **4j,** with IC_50_ values between 18.6 and 31.5 µM (Table 3).

The experimental data revealed interesting insights into the structure-activity relationship. Incorporation of different electron-donating and -withdrawing groups on the indole ring has a significant impact on the activity of the derivatives. The nitro group substitution either had a negligible or nonexistent inhibitory effect on the cancer cell lines. The methoxy group substitution exerted a positive influence and was more active compared to the unsubstituted compound. For halogens, the fluorine substitution was considerably more active than the bromine substitution. This improved inhibitory effect due to the bio-isosteric substitution of hydrogen with fluorine may be attributable to changed pharmacokinetic features since fluorine is a strongly electron-withdrawing group that reduces the potential for oxidative metabolism.

### 2.3. Molecular Docking Studies

**Figure 2:**
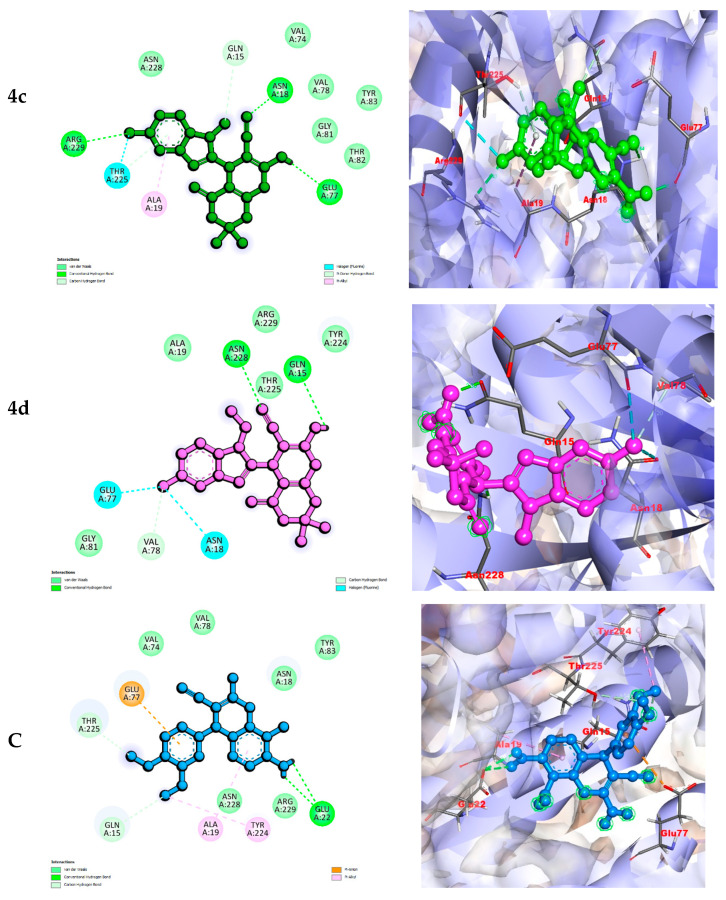
In silico binding interactions of the active derivatives **4c** (green) and **4d** (pink) with tubulin protein (6JCJ). Crolibulin (**C**, blue) was used as a positive control in the docking experiment.

Tubulin protein plays a dynamic role in critical cellular functions and is a molecular target in the design of new anticancer agents [18]. The design of new tubulin-targeting agents is well researched, and a few of the drug candidates are in the clinical stages of development [19]. The cytotoxicity experiments showed that the novel indole-tethered chromene derivatives **4c** and **4d** showed good anti-proliferative activity. To understand their binding affinity towards tubulin, we docked the derivatives **4c** and **4d** with the target protein tubulin (6JCJ), and crolibulin was used as a positive control. The studies revealed that novel indole-tethered chromene derivatives exhibited better binding affinity toward the tubulin protein than the control (Figure 2). The derivative **4c,** with fluorine and methyl substitutions on the indole ring, displayed the best interaction with a binding energy score of −6.4 kcal/mol (Table 4). The key interaction resulted from the formation of five hydrogen bonds with different amino acid residues of the target protein, namely asn18, arg229, glu77, gln15, and thr225. The bond lengths of these hydrogen bonds were between 1.93 and 3.42 Å. The other non-covalent interactions were van der Waals with asn228, val74, val78, gly81, thr82, and tyr83 residues, and single pi-alkyl and halogen interactions with ala19 and thr225 residues, respectively. The other derivative, **4d**, with fluorine and an ethyl group on the indole ring, also showed binding affinity towards the target protein with a slightly lowered binding affinity score of −6.1 kcal/mol. It primarily resulted from three hydrogen bond formations with amino acid residues: asn228, gln15, and val78, with bond lengths between 2.36 and 3.20 Å. The non-covalent interactions include five van der Waals interactions with the residues, gly81, ala19, arg229, thr225, and tyr224; and two halogen interactions with the glu77 and asn18 residues. In contrast, the control showed the least binding affinity towards the target protein in comparison to the two derivatives. A binding energy score of −5.6 kcal/mol was observed for the control with four hydrogen bond formations (2.56–3.68 Å), with the residues, glu22, glu22, gln15, and thr225. In addition to this, six van der Waals interactions with val74, val78, asn18, tyr83, arg229, and asn228 residues in addition to pi-alkyl as well as pi-anion interactions with ala19 and tyr224, respectively, were also observed. The docking study revealed that the derivatives **4c** and **4d** displayed enhanced binding affinity towards the tubulin protein compared to the control.

### 2.4. Molecular Dynamics Simulations

**Figure 3:**
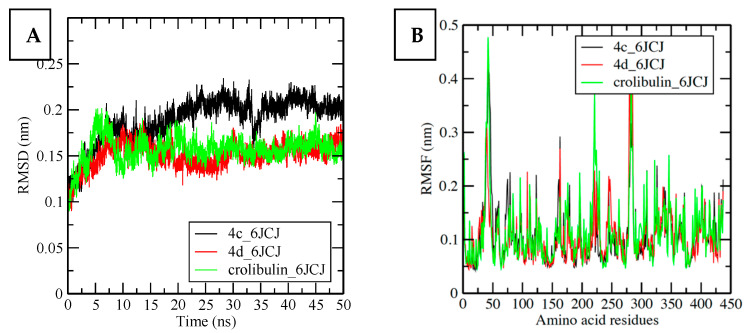

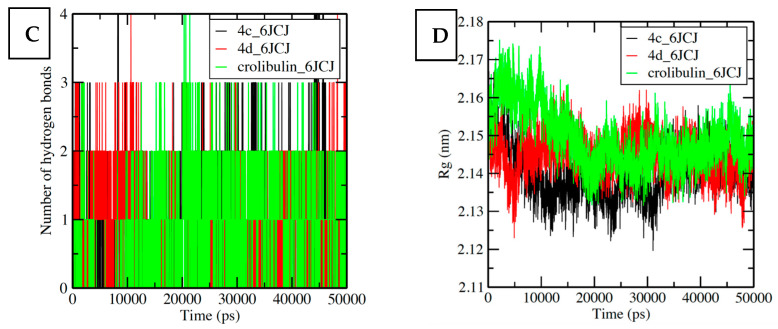
Molecular dynamics simulations of the 50 ns period: (**A**) RMSD plot of ligands (**4c**, **4d,** and crolibulin)-tubulin (6JCJ) complexes. (**B**) RMSF plot of the selected complexes with fluctuation per residue. (**C**) The hydrogen bond plot shows the number of formations of hydrogen bonds between ligands and receptors. (**D**) Radius of gyration (Rg) plot of the thre complexes.

The molecular docking studies exhibited a stronger degree of interaction between the compounds (**4c** and **4d**) and tubulin protein than with crolibulin, as implied from the binding energy values. The stability of these ligand-protein complexes was determined by molecular dynamics simulation studies to further understand their affinity for the target tubulin protein. Structural stability and similarity play a role in the formation of ligand-protein complexes, and the root mean square deviation (RMSD) values provide insights into the stability of complexes. Lower values indicate enhanced stability. The average RMSD values for **4c**, **4d**, and crolibulin (ligand)-tubulin complexes were between 0.1 and 0.2 nm (Figure 3A). Interestingly, it was observed that **4d** and crolibulin simulations with tubulin showed similar stable patterns with an average value of approximately 0.15 nm. The average fluctuation of the amino acid residues of the target protein during binding with the ligands was studied. The RMSF fluctuation plot values ranged between 0.1 and 0.5 nm for complexes, and the observed average value was ca. 0.1 nm except for some major fluctuations at the 24–50 and 275–280 amino acid residue regions. The **4c**-tubulin complex showed a maximum spike at approximately 25–50 and 225–230 amino acid regions (Figure 3B). The complex compactness profile is given the radius of gyration, and the observed values of Rg were between 1.14 and 1.15 nm for **4c**, **4d**, and crolibulin-tubulin complexes (Figure 3C). The hydrogen bond plot showed the formation of 1–4 hydrogen bonds during the simulation study (Figure 3D).

### 2.5. ADME, Drug-Likeness Analysis

Computational analysis is an effective and attractive alternative to experimental validation that predicts ADME profiles and drug-likeness properties. It helps to reduce costs, addresses the ethical concerns of using both humans and animals in trials [20], and identifies molecules with desired properties that could be taken forward for experimental testing. We analysed the ADME and drug-likeness properties of indole-tethered chromenes **4a**–**j** using computational tools. The study showed that all the derivatives satisfy Lipinski’s rule of five, indicating good oral bioavailability as indicated by the values of 0.55–0.56 (Table 5). Interestingly, the other drug-likeliness filters, namely Ghose, Veber, Egan, and Muegge, were also not violated. Additionally, all the compounds were found to be lipophilic as indicated by positive log P values, which are less than five. Similar to the control, all the derivatives were non-permeable across the blood-brain barrier with high GI absorption, except **4f**. All the derivatives displayed good skin penetration, with log Kp values ranging from −7.02 to −6.2 cm/s, indicating their transport through the mammalian epidermis. Most of the derivatives were moderately water-soluble, as indicated by values ranging from −4.04 to −6.31 for all the models considered for water solubility, and the topological polar surface area values were in the range of 81–111. Cytochrome enzymes play a significant role in the metabolism of drugs and other xenobiotics and are a key parameter in drug metabolism studies [21]. All the derivatives **4a–4j** displayed inhibition of CYP 2C19, 2C9, and 3A4 isoforms but no inhibition of CYP 2D6 (Table 5). For the isoform 1A2, few compounds **4a**, **4b**, **4c,** and **4i** showed inhibition and the compounds **4d**, **4e**, **4f**, **4g**, **4h,** and **4j** showed no inhibition. The complete physiochemical, lipophilicity, water solubilities, pharmacokinetics, and drug-likeness profiles of these derivatives are provided in the Supplementary Information.

## 3. Materials and Methods

General Information: Various analytical techniques were used to characterise the novel compounds. The ^13^C and ^1^H NMR spectra were acquired by means of a Bruker DRX 400 spectrometer with 100 MHz and 400 MHz resolutions in DMSO-d_6_ using TMS as the internal standard. A mass spectrum was obtained using an Agilent-LCMS device (Agilent, Santa Clara, CA, USA). KBr pellets were used to record FT-IR spectra on a VERTEX 70 Bruker (Bruker, Rosenheim, Germany). All the melting points were measured in an open capillary tube immersed in a sulphuric acid bath and uncorrected. Without further purification, all the solvents and reagents that were available commercially were put to use.

### 3.1. Chemical Synthesis

#### 3.1.1. Method of the Synthesis of Novel Indole-Tethered Chromenes **4a**–**j**

A round-bottomed flask was taken and charged with indole-3-carbaldehydes **1a**–**j** (10 mmol), 5,5-dimethyl cyclohexane-1,3-dione **2** (10 mmol), and malononitrile **3** (10 mmol) in 50 mL of ethanol, and to it, 0.3 eq of DBU was added. The reaction mixture was heated to 60–65 °C for 60–90 min and monitored by thin-layer chromatography. After the completion of the reaction, the reaction mixture was cooled, and cold water was added and stirred for 10–15 min. The separated solid was filtered, washed with 50 mL of water, and vacuum-dried at 60–65 °C for 8–10 h. Using ethanol as the solvent, the crude product was refined by recrystallization to generate the required compounds **4a**–**j** with an 80–85% yield.

#### 3.1.2. 2-Amino-7,7-dimethyl-4-(1-methyl-1H-indol-2-yl)-5-oxo-5,6,7,8-tetrahydro-4H-chromene-3-carbonitrile (**4a**)

The product **4a** was prepared from substrates **1a**, **2**, and **3** using the general procedure. M.P: 253–255 °C; yield: 85%; IR (KBr): 3345 (broad, -NH), 2105 (C=N), 1720 (C=O) cm^−1^; ^1^H NMR δ (400 MHz; CDCl_3_): 0.9 (s, 6H, -CH_3_), 1.8 and 2.2 (s, 4H, -CH_2_), 3.6 (s, 3H, -CH_3_), 4.2 (s, 1H, -CH), 7.2–8.2 (m, 7H, Ar-H and -NH_2_); ^13^C NMR δ (100 MHz; CDCl_3_): 19.1, 20.2, 20.3, 29.6, 32.0, 49.3, 60.8, 121.5, 127.8, 128.3, 128.6, 130.0, 130.2, 132.7, 138.7, 140.8, 141.0, 142.7, 143.8, 145.1, and 170.8; and [M+H^+^]: 348.

#### 3.1.3. 2-Amino-4-(1-ethyl-1H-indol-2-yl)-7,7-dimethyl-5-oxo-5,6,7,8-tetrahydro-4H-chromene-3-carbonitrile (**4b**) 

The product **4b** was prepared from substrates **1b**, **2**, and **3** using the general procedure. M.P: 267–269 °C; yield: 83%; IR (KBr): 3341 (broad, -NH), 2113 (C=N), 1712 (C=O) cm^−1^; ^1^H NMR δ (400 MHz; CDCl_3_): 0.9 (s, 6H, -CH_3_), 1.2 (q, 3H, -CH_3_), 1.9 and 2.3 (s, 4H, -CH_2_), 3.8 (t, 2H, -CH_2_), 4.2 (s, 1H, -CH), 7.2–8.2 (m, 7H, Ar-H and -NH_2_); ^13^C NMR δ (100 MHz; CDCl_3_): 19.2, 20.3, 20.4, 29.5, 32.1, 49.2, 60.9, 122.4, 127.9, 129.4, 129.9, 130.1, 130.4, 132.6, 138.8, 140.9, 141.3, 142.9, 143.9, 145.4, and 170.1; and [M+H^+^]: 362. 

#### 3.1.4. 2-Amino-4-(5-fluoro-1-methyl-1H-indol-2-yl)-7,7-dimethyl-5-oxo-5,6,7,8-tetrahydro-4H-chromene-3-carbonitrile (**4c**) 

The product **4c** was prepared from substrates **1c**, **2**, and **3** using the general procedure. M.P: 263–265 °C; yield: 84%; IR (KBr): 3349 (broad, -NH), 2117 (C=N), 1713 (C=O) cm^−1^; ^1^H NMR δ (400 MHz; CDCl_3_): 0.9 (s, 6H, -CH_3_), 1.8 and 2.2 (s, 4H, -CH_2_), 3.6 (s, 3H, -CH_3_), 4.2 (s, 1H, -CH), 7.2–8.2 (m, 6H, Ar-H and -NH_2_); ^13^C NMR δ (100 MHz; CDCl_3_): 19.1, 20.1, 20.4, 29.4, 32.0, 49.2, 60.8, 123.2, 125.9, 128.7, 128.9, 130.0, 131.3, 132.8, 135.1, 139.4, 145.9, 146.1, and 168.2; and [M+H^+^]: 366. 

#### 3.1.5. 2-Amino-4-(1-ethyl-5-fluoro-1H-indol-2-yl)-7,7-dimethyl-5-oxo-5,6,7,8-tetrahydro-4H-chromene-3-carbonitrile (**4d**) 

The product **4d** was prepared from substrates **1d**, **2**, and **3** using the general procedure. M.P: 281–283 °C; yield: 84%; IR (KBr): 3338 (broad, -NH), 2213 (C=N), 1711 (C=O) cm^−1^; ^1^H NMR δ (400 MHz; CDCl_3_): 0.9 (s, 6H, -CH_3_), 1.2 (q, 3H, -CH_3_), 1.8 and 2.2 (s, 4H, -CH_2_), 3.8 (t, 2H, -CH_2_), 4.2 (s, 1H, -CH), 7.2–8.2 (m, 6H, Ar-H and -NH_2_); ^13^C NMR δ (100 MHz; CDCl_3_): 14.1, 19.0, 20.6, 20.7, 29.2, 42.5, 48.8, 60.6, 121.5, 127.8, 128.3, 128.6, 130.0, 132.7, 138.7, 140.8, 141.0, 142.7, and 169.5; and [M+H^+^]: 380. 

#### 3.1.6. 2-Amino-7,7-dimethyl-4-(1-methyl-5-nitro-1H-indol-2-yl)-5-oxo-5,6,7,8-tetrahydro-4H-chromene-3-carbonitrile (**4e**) 

The product **4e** was prepared from substrates **1e**, **2**, and **3** using the general procedure. M.P: 245–247 °C; yield: 85%; IR (KBr): 3342 (broad, -NH), 2119 (C=N), 1709 (C=O) cm^−1^; ^1^H NMR δ (400 MHz; CDCl_3_): 0.9 (s, 6H, -CH_3_), 1.8 and 2.2 (s, 4H, -CH_2_), 3.7 (s, 3H, -CH_3_), 4.3 (s, 1H, -CH), 7.2–8.3 (m, 6H, Ar-H and -NH_2_); ^13^C NMR δ (100 MHz; CDCl_3_): 19.3, 20.0, 20.5, 29.5, 31.9, 48.3, 60.9, 122.3, 125.6, 129.8, 129.9, 131.0, 131.5, 133.8, 135.4, 139.3, 144.8, 146.3, and 169.1; and [M+H^+^]: 393. 

#### 3.1.7. 2-Amino-4-(1-ethyl-5-nitro-1H-indol-2-yl)-7,7-dimethyl-5-oxo-5,6,7,8-tetrahydro-4H-chromene-3-carbonitrile (**4f**) 

The product **4f** was prepared from substrates **1f**, **2**, and **3** using the general procedure. M.P: 272–274 °C; yield: 84%; IR (KBr): 3341 (broad, -NH), 2186 (C=N), 1709 (C=O) cm^−1^; ^1^H NMR δ_H_ (400 MHz; CDCl_3_): 0.9 (s, 6H, -CH_3_), 1.1 (q, 3H, -CH_3_), 1.9 and 2.2 (s, 4H, -CH_2_), 3.9 (t, 2H, -CH_2_), 4.3 (s, 1H, -CH), 7.2–8.4 (m, 6H, Ar-H and -NH_2_); ^13^C NMR δ (100 MHz; CDCl_3_): 14.3, 19.1, 21.0, 21.9, 28.1, 42.6, 48.9, 60.7, 121.4, 126.9, 128.4, 128.9, 131.1, 132.6, 139.8, 141.9, 142.8, 142.9, and 169.6; and [M+H^+^]: 407. 

#### 3.1.8. 2-Amino-4-(5-methoxy-1-methyl-1H-indol-2-yl)-7,7-dimethyl-5-oxo-5,6,7,8-tetrahydro-4H-chromene-3-carbonitrile (**4g**) 

The product **4g** was prepared from substrates **1g**, **2**, and **3** using the general procedure. M.P: 254–256 °C; yield: 83%; IR (KBr): 3332 (broad, -NH), 2118 (C=N), 1706 (C=O) cm^−1^; ^1^H NMR δ (400 MHz; CDCl_3_): 0.9 (s, 6H, -CH_3_), 1.8 and 2.2 (s, 4H, -CH_2_), 3.3 (s, 3H, -OCH_3_), 3.8 (s, 3H, -CH_3_), 4.2 (s, 1H, -CH), 7.4–8.3 (m, 6H, Ar-H and -NH_2_); ^13^C NMR δ (100 MHz; CDCl_3_): 19.1, 20.1, 20.3, 29.1, 31.6, 49.1, 54.3, 60.8, 123.8, 128.7, 128.8, 129.1, 130.3, 131.1, 132.8, 134.6, 135.0, 138.7, 140.2, 145.8, 145.9, and 169.2; and [M+H^+^]: 378. 

#### 3.1.9. 2-Amino-4-(1-ethyl-5-methoxy-1H-indol-2-yl)-7,7-dimethyl-5-oxo-5,6,7,8-tetrahydro-4H-chromene-3-carbonitrile (**4h**)

The product **4h** was prepared from substrates **1h**, **2**, and **3** using the general procedure. M.P: 292–294 °C; yield: 82%; IR (KBr): 3332 (broad, -NH), 2188 (C=N), 1703 (C=O) cm^−1^; ^1^H NMR δ (400 MHz; CDCl_3_): 0.9 (s, 6H, -CH_3_), 1.2 (q, 3H, -CH_3_), 1.8 and 2.2 (s, 4H, -CH_2_), 3.3 (s, 3H, -OCH_3_), 3.8 (t, 2H, -CH_2_), 4.2 (s, 1H, -CH), 7.3–8.4 (m, 6H, Ar-H and -NH_2_); ^13^C NMR δ (100 MHz; CDCl_3_): 14.0, 18.9, 21.1, 21.8, 28.2, 41.7, 48.8, 61.7, 120.3, 124.8, 126.5, 128.8, 130.2, 132.7, 138.8, 140.8, 142.9, 143.9, and 170.0; and [M+H^+^]: 392. 

#### 3.1.10. 2-Amino-4-(5-bromo-1-methyl-1H-indol-2-yl)-7,7-dimethyl-5-oxo-5,6,7,8-tetrahydro-4H-chromene-3-carbonitrile (**4i**) 

The product **4i** was prepared from substrates **1i**, **2**, and **3** using the general procedure. M.P: 270–272 °C; yield: 81%; IR (KBr): 3333 (broad, -NH), 2119 (C=N), 1701 (C=O) cm^−1^; ^1^H NMR δ (400 MHz; CDCl_3_): 0.9 (s, 6H, -CH_3_), 1.8 and 2.2 (s, 4H, -CH_2_), 3.7 (s, 3H, -CH_3_), 4.2 (s, 1H, -CH), 7.4–8.4 (m, 6H, Ar-H and -NH_2_); ^13^C NMR δ (100 MHz; CDCl_3_): 18.9, 20.2, 20.4, 29.0, 30.5, 49.2, 53.2, 60.5, 121.6, 128.6, 128.9, 129.0, 130.2, 132.1, 132.7, 133.7, 135.1, 138.6, 141.2, 145.9, 145.8, and 169.6; and [M^+^ and M^+2^]: 425 and 427. 

#### 3.1.11. 2-Amino-4-(5-bromo-1-ethyl-1H-indol-2-yl)-7,7-dimethyl-5-oxo-5,6,7,8-tetrahydro-4H-chromene-3-carbonitrile (**4j**) 

The product **4j** was prepared from the substrates **1j**, **2**, and **3** using the general procedure. M.P: 280–282 °C; yield: 82%; IR (KBr): 3339 (broad, -NH), 2189 (C=N), 1709 (C=O) cm^−1^; ^1^H NMR δ (400 MHz; CDCl_3_): 0.9 (s, 6H, -CH_3_), 1.2 (q, 3H, -CH_3_), 1.8–2.2 (s, 4H, -CH_2_), 3.8 (t, 2H, -CH_2_), 4.3 (s, 1H, -CH), 7.2–8.4 (m, 6H, Ar-H and -NH_2_); ^13^C NMR δ (100 MHz; CDCl_3_): 14.1, 18.8, 20.3, 21.9, 28.3, 41.6, 48.7, 60.6, 121.5, 124.9, 126.6, 128.9, 130.1, 132.6, 138.9, 140.6, 142.8, 143.8, and 170.2; and [M^+^ and M^+2^]: 439 and 441.

### 3.2. Assay of Cytotoxicity

The anticancer efficacy of the newly synthesised indole-tethered chromenes **4a**–**j** was determined employing the standard MTT assay [22] on a panel of three different human cancer cell lines: A549 (lung carcinoma), MCF-7 (breast carcinoma), and PC-3 (prostate carcinoma) cells. DMSO was used to dissolve the synthetic compounds and doxorubicin (the control). The tumour cells were seeded at a density of 1.6×10^5^ cells per 100 µL of DMEM cell culture medium and cultured for 24 h before adding various concentrations of test compounds in 96 well plates. The cells were subsequently treated for 48 h using produced chemicals of varying concentrations. After incubation, PBS (200 µL) was used to wash the wells and incubate with a 10% MTT solution at 37 °C for two hours. The optical density at 570 nm was determined by a multimode reader (Tecan Infinite 200 PRO, Switzerland).

### 3.3. Computational Studies

#### 3.3.1. Molecular Modeling

The docking study was conducted between the derivatives (**4c**, **4d**) and tubulin proteins using the PyRx tool, version 0.8, which utilises the scoring functions of AutoDock. The crystal structure of tubulin protein in complex with crolibulin was taken from the RCSB PDB (ID: 6JCJ). Crolibulin, HETATOM, and water molecules were removed from the tubulin protein, and the CHARMM force field was used for energy minimization. The docking was performed within the grid box of 25x25x25 Å, and the grid centre point coordinates X, Y, and Z were set as −24.187, −79.493, and 50.488, respectively. 

#### 3.3.2. Molecular Dynamics Simulations

MDS of ligand-receptor complexes (**4c**, **4d,** crolibulin with tubulin-PDB ID:6JCJ) was performed for fifty nanoseconds (ns) using the GROningen Machine for Chemical Simulations (GROMACS) tool 2018 version [23]. The pdb2gmx module was used to generate the required receptor molecule, i.e., the 6JCJ topology file, followed by CHARMM27 all-atom force field selection and generation of topology files for ligands (**4c**, **4d,** and crolibulin). The NVT and NPT ensembles provided control over temperature and pressure, resulting in constancy and stabilisation of the system. Finally, after a successful 50 ns simulation run, trajectory files and graphical plots were generated by the Xmgrace program.

#### 3.3.3. ADME and Drug-Likeness Predictions

ADME and drug-likeness properties were analysed computationally using the Swiss ADME programme [24].

## 4. Conclusions

The presence of heterocycles in two-thirds of the anticancer medications approved by the FDA indicates their significance in cancer research and their critical role in the fight against cancer. Multi-component reactions (MCRs) have played a significant role in chemistry and the pharmaceutical industries as they allow for the synthesis of functionalized heterocyclic molecules using a single operating approach and easily accessible precursors. The key benefits of this present MCR protocol are its atom economy, short reaction time, mild reaction condition with a simple workup, and satisfactory yields. Cytotoxicity screening indicated that most synthesised compounds showed promising anticancer potential against the A549, PC-3, and MCF-7 cancer cell lines. Derivatives **4d** and **4c** were the most promising compounds in the series and displayed single-digit IC_50_ values. Docking experiments demonstrated that the active derivatives have good binding affinity towards the tubulin protein, better than the control. The molecular dynamics simulation studies further validated the binding affinity of the potent derivatives to the tubulin protein. The ADME predictions were encouraging, and the derivatives follow all five drug-likeness filters. Overall, these synthesised compounds may serve as initial hits, and further investigation is needed to develop them as potential leads.

## Figures and Tables

**Table 1:** Optimization of the model reaction ^a^.

Entry	Solvent	Temperature (°C)	Catalyst (0.3 eq)	Time (min)	Yield (%)
1	Ethanol	60–65	pyridine	120	65
2	Methanol	60–65	pyridine	120	70
3	Acetonitrile	60–65	pyridine	240	72
4	DMF	60–65	pyridine	180	68
5	Ethanol	60–65	DBU	60	85
6	Methanol	60–65	DBU	100	80
7	Acetonitrile	60–65	DBU	120	75
8	DMF	60–65	DBU	120	75
9	Ethanol	70–75	DBU	55	83
10	Ethanol	50–55	DBU	180	85

^a^ One mmol of substrates **1a**, **2**, and **3** in the presence of 0.3 equivalent of catalyst.

**Table 2:** Optimization of the amount of DBU ^a^.

Entry	Solvent	Temperature in °C	Amount of DBU	Time (min)	Yield (%)
1	Ethanol	60–65	0.1 eq	240	85
2	Ethanol	60–65	0.3 eq	60	85
3	Ethanol	60–65	0.5 eq	50	80

^a^ One mmol of substrates **1a**, **2**, and **3** in the presence of varying amounts of DBU.

**Table 3:** Cytotoxicity data of novel indole-tethered chromenes **4a**–**j** ^a^.

Compound	IC_50_ Values		
	A549	PC-3	MCF-7
**4a**	19.6 ± 0.11	18.6 ± 0.19	19.2 ± 0.23
**4b**	22.3 ± 0.18	21.4 ± 0.25	20.9 ± 0.31
**4c**	8.1 ± 0.25	9.1 ± 0.39	8.4 ± 0.17
**4d**	7.9 ± 0.16	8.9 ± 0.22	8.6 ± 0.22
**4e**	na	na	58.9 ± 0.48
**4f**	na	na	62.8 ± 0.34
**4g**	10.5 ± 0.17	11.2 ± 0.28	12.6 ± 0.32
**4h**	11.9 ± 0.29	12.7 ± 0.33	12.1 ± 0.26
**4i**	25.3 ± 0.25	29.8 ± 0.34	26.1 ± 0.19
**4j**	30.2 ± 0.22	31.5 ± 0.27	29.4 ± 0.21
Dox	0.8 ± 0.06	0.6 ± 0.04	0.7± 0.08

^a^ IC_50_ values are reported in μM as the mean of triplicates with a standard deviation. Doxorubicin as a positive control.

**Table 4:** Docking analysis of the covalent and non-covalent interactions of the derivatives (**4c** and **4d**) with the target protein, tubulin (6JCJ).

Compd	Binding Affinity (Kcal/mol)	Hydrogen Bond	Hydrogen Bond Length (Angstrom)	Van der Waals Interaction	Other Interactions
**4c**	−6.4	A:ASN18:HD22-:UNL1:N2	2.64	ASN228,VAL74,VAL78,GLY81,THR82,TYR83	Pi-Alkyl = ALA19 Halogen = THR225
		A:ARG229:HH11-:UNL1:F1	2.47		
		:UNL1:H15-A:GLU77:O	1.93		
		:UNL1:C18-A:GLN15:OE1	3.42		
		A:THR225:HG1-:UNL1	3.18		
**4d**	−6.1	A:ASN228:HD21-:UNL1:N2	2.36	GLY81,ALA19,ARG229,THR225,TYR224	Halogen GLU77,ASN18
		:UNL1:H15-A:GLN15:OE1	2.36		
		A:VAL78:CA–:UNL1:F1	3.20		
**Control**	−5.6	:UNL1:H12–A:GLU22:OE1	2.71	VAL74,VAL78,ASN18,TYR83,ARG229,ASN228	Pi-Alkyl = ALA19Pi-Anion = TYR224
		:UNL1:H13–A:GLU22:OE1	2.56		
		:UNL1:C1–A:GLN15:OE1	3.68		
		:UNL1:C8–A:THR225:OG1	3.54		

**Table 5:** Key computational calculations of derivatives **4a**–**j**.

Compd	Inhibition of Cytochrome Enzymes					TPSA Value	Log Kp (cm/s)	Follow Drug-likeness Filter
	CYP 1A2	CYP 2C19	CYP 2C9	CYP 2D6	CYP 3A4			
**4a**	Y	Y	Y	N	Y	81.4	−6.33	5
**4b**	Y	Y	Y	N	Y	81.4	−6.2	5
**4c**	Y	Y	Y	N	Y	81.4	−6.37	5
**4d**	N	Y	Y	N	Y	81.4	−6.24	5
**4e**	N	Y	Y	N	Y	126.86	−6.73	5
**4f**	N	Y	Y	N	Y	126.86	−6.6	5
**4g**	N	Y	Y	N	Y	90.27	−6.54	5
**4h**	N	Y	Y	N	Y	90.27	−6.41	5
**4i**	Y	Y	Y	N	Y	81.08	−6.32	5
**4j**	N	Y	Y	N	Y	81.04	−6.2	5
**Control**	Y	Y	Y	Y	Y	129.54	−7.02	5

## Data Availability

Data is contained within the article and Supplementary Material.

## Supplementary Materials

The following supporting information can be downloaded at: https://www.mdpi.com/article/10.3390/ph16030333/s1.
