# Supplementary material for: Novel Indole-Tethered Chromene Derivatives: Synthesis, Cytotoxic Properties, and Key Computational Insights
Source: Pharmaceuticals (Basel). 2023 Feb 22;16(3):333. doi: 10.3390/ph16030333 (PMC10051285; doi:10.3390/ph16030333)
Supplement: Supplementary file 1 [file pharmaceuticals-16-00333-s001.zip › pharmaceuticals-2160267-supplementary.pdf]

Supporting information For

Novel indole fused chromene derivatives: Synthesis, cytotoxic properties and key computational insights

M. Shaheer Malik[a]*†, Hissana Ather[b]*†, Shaik Mohammad Asif Ansari[c], Ayesha Siddiqua[c], Qazi Mohammad Sajid Jamal[d], Ali H. Alharbi,[d] Munirah M. Al-Rooqi[a] , Rabab S. Jassas[e], Essam M. Hussein[a,f], Ziad Moussa[g], Rami J. Obaid[a] and Saleh A. Ahmed[a,e]*

[a]Department of Chemistry, Faculty of Applied Sciences, Umm Al-Qura University, Makkah 21955, Saudi Arabia

[b]Department of Pharmaceutical Chemistry, College of Pharmacy, King Khalid University (KKU), Abha 62529, Saudi Arabia

[c]Department of Clinical Pharmacy, College of Pharmacy, King Khalid University (KKU), Abha 62529, Saudi Arabia.

[d]Department of Health Informatics, College of Public Health and Health Informatics, Qassim University, Al Bukayriyah, Saudi Arabia

[e]Department of Chemistry, Jamoum University College, Umm Al-Qura University, 21955 Makkah, Saudi Arabia

[f]Department of Chemistry, Faculty of Science, Assiut University, 71516 Assiut, Egypt

[g]Department of Chemistry, College of Science, United Arab Emirates University, 15551 Al Ain, United Arab Emirates

Correspondence: may be addressed to:

M. Shaheer Malik, E-mail address: msmalik@uqu.edu.sa, Hissana Ather, E-mail address: hissana@kku.edu.sa, Saleh A. Ahmed, E-mail address: saahmed@uqu.edu.sa

- **Table S1**: Physicochemical properties of compounds **4a-j**
- **Table S2**: Lipophilicity and water solubility of compounds **4a-j**
- **Table S3**: Some other water solubility parameters of compounds **4a-j**
- **Table S4**: Pharmacokinetics parameters of compounds **4a-j**
- **Table S5**: Drug likeness and medicinal chemistry of compounds **4a-j**
- Spectra

Table S1: Physicochemical properties of compounds **4a-j**

Molecule	#Heavy atoms	#Aromatic heavy atoms	Fraction Csp3	#Rotatable bonds	#H-bond acceptors	#H-bond donors	MR	TPSA
4a	26	9	0.33	1	3	1	99.35	81.04
4b	27	9	0.36	2	3	1	104.16	81.04
4c	27	9	0.33	1	4	1	99.31	81.04
4d	28	9	0.36	2	4	1	104.11	81.04
4e	29	9	0.33	2	5	1	108.17	126.86
4f	30	9	0.36	3	5	1	112.98	126.86
4g	28	9	0.36	2	4	1	105.84	90.27
4h	29	9	0.39	3	4	1	110.65	90.27
4i	27	9	0.33	1	3	1	107.05	81.04
4j	28	9	0.36	2	3	1	111.86	81.04
CTRL	26	12	0.17	3	4	3	101.36	129.54

Table S2: Lipophilicity and water solubility of compounds **4a-j**

Molecule	iLOGP	XLOGP3	WLOGP	MLOGP	Silicos-IT Log P	Consensus Log P	ESOL Log S	ESOL Solubility (mg/ml)
**4a**	2.81	2.94	3.63	1.57	3.01	2.79	-4.04	3.20E-02
**4b**	2.97	3.24	4.11	1.79	3.4	3.1	-4.24	2.10E-02
**4c**	2.94	3.04	4.19	1.95	3.43	3.11	-4.2	2.30E-02
**4d**	3.11	3.34	4.67	2.17	3.82	3.42	-4.4	1.50E-02
**4e**	2.57	2.77	3.54	1.46	0.85	2.24	-4.12	3.01E-02
**4f**	2.73	3.07	4.02	1.68	1.25	2.55	-4.32	1.95E-02
**4g**	3.04	2.91	3.64	1.24	3.07	2.78	-4.12	2.87E-02
**4h**	3.03	3.21	4.12	1.46	3.47	3.06	-4.32	1.87E-02
**4i**	3.2	3.63	4.39	2.17	3.69	3.42	-4.95	4.78E-03
**4j**	3.38	3.93	4.87	2.38	4.08	3.73	-5.15	3.10E-03
CTRL	26	12	0.17	3	4	3	101.36	129.54

Table S3: Some other water solubility parameters of compounds **4a-j**

**Molecule**	**ESOL Class**	**Ali Log S**	**Ali Solubility (mg/ml)**	**Ali Solubility (mol/l)**	**Ali Class**	**Silicos-IT LogSw**	**Silicos-IT Solubility (mg/ml)**	**Silicos-IT Solubility (mol/l)**	**Silicos-IT class**
**4a**	Moderately soluble	-4.3	1.73E-02	4.97E-05	Moderately soluble	-5.13	2.55E-03	7.35E-06	Moderately soluble
**4b**	Moderately soluble	-4.62	8.77E-03	2.43E-05	Moderately soluble	-5.53	1.07E-03	2.96E-06	Moderately soluble
**4c**	Moderately soluble	-4.41	1.43E-02	3.91E-05	Moderately soluble	-5.4	1.45E-03	3.97E-06	Moderately soluble
**4d**	Moderately soluble	-4.72	7.25E-03	1.91E-05	Moderately soluble	-5.79	6.08E-04	1.60E-06	Moderately soluble
**4e**	Moderately soluble	-5.09	3.19E-03	8.13E-06	Moderately soluble	-4.48	1.30E-02	3.31E-05	Moderately soluble
**4f**	Moderately soluble	-5.4	1.61E-03	3.97E-06	Moderately soluble	-4.87	5.44E-03	1.34E-05	Moderately soluble
**4g**	Moderately soluble	-4.47	1.29E-02	3.42E-05	Moderately soluble	-5.24	2.17E-03	5.75E-06	Moderately soluble
**4h**	Moderately soluble	-4.78	6.53E-03	1.67E-05	Moderately soluble	-5.63	9.09E-04	2.32E-06	Moderately soluble
**4i**	Moderately soluble	-5.02	4.07E-03	9.55E-06	Moderately soluble	-5.92	5.10E-04	1.20E-06	Moderately soluble

**4j**	Moderately soluble	-5.33	2.05E-03	4.66E-06	Moderately soluble	-6.31	2.14E-04	4.86E-07	Poorly soluble
CTRL	Moderately soluble	-4.94	4.81E-03	1.15E-05	Moderately soluble	-5.25	2.36E-03	5.65E-06	Moderately soluble

Table S4: Pharmacokinetics parameters of compounds **4a-j**

**Molecule**	GI absorption	BBB permeant	Pgp substrate	CYP1A2 inhibitor	CYP2C19 inhibitor	CYP2C9 inhibitor	CYP2D6 inhibitor	CYP3A4 inhibitor	log Kp (cm/s)
**4a**	High	No	Yes	Yes	Yes	Yes	No	Yes	-6.33
**4b**	High	No	Yes	Yes	Yes	Yes	No	Yes	-6.2
**4c**	High	No	Yes	Yes	Yes	Yes	No	Yes	-6.37
**4d**	High	No	Yes	No	Yes	Yes	No	Yes	-6.24
**4e**	High	No	Yes	No	Yes	Yes	No	Yes	-6.73
**4f**	Low	No	Yes	No	Yes	Yes	No	Yes	-6.6
**4g**	High	No	Yes	No	Yes	Yes	No	Yes	-6.54
**4h**	High	No	Yes	No	Yes	Yes	No	Yes	-6.41
**4i**	High	No	Yes	Yes	Yes	Yes	No	Yes	-6.32
**4j**	High	No	Yes	No	Yes	Yes	No	Yes	-6.2
CTRL	High	No	Yes	Yes	Yes	Yes	Yes	Yes	-7.02

Table S5: Drug likeness and medicinal chemistry of compounds **4a-j**

**Molecule**	Lipinski #violations	Ghose #violations	Veber #violations	Egan #violations	Muegge #violations	Bioavailability Score	PAINS #alerts	Brenk #alerts	Leadlikeness #violations	Synthetic accesibility
**4a**	0	0	0	0	0	0.56	0	0	0	4.19
**4b**	0	0	0	0	0	0.56	0	0	1	4.29
**4c**	0	0	0	0	0	0.56	0	0	1	4.18
**4d**	0	0	0	0	0	0.56	0	0	1	4.3
**4e**	0	0	0	0	0	0.56	0	2	1	4.29
**4f**	0	0	0	0	0	0.56	0	2	1	4.39
**4g**	0	0	0	0	0	0.56	0	0	1	4.27
**4h**	0	0	0	0	0	0.56	0	0	1	4.38
**4i**	0	0	0	0	0	0.56	0	0	2	4.19
**4j**	0	0	0	0	0	0.56	0	0	2	4.29
CTRL	0	0	0	0	0	0.55	0	1	1	4.14

Spectroscopic Characterization of the Synthesized Compounds

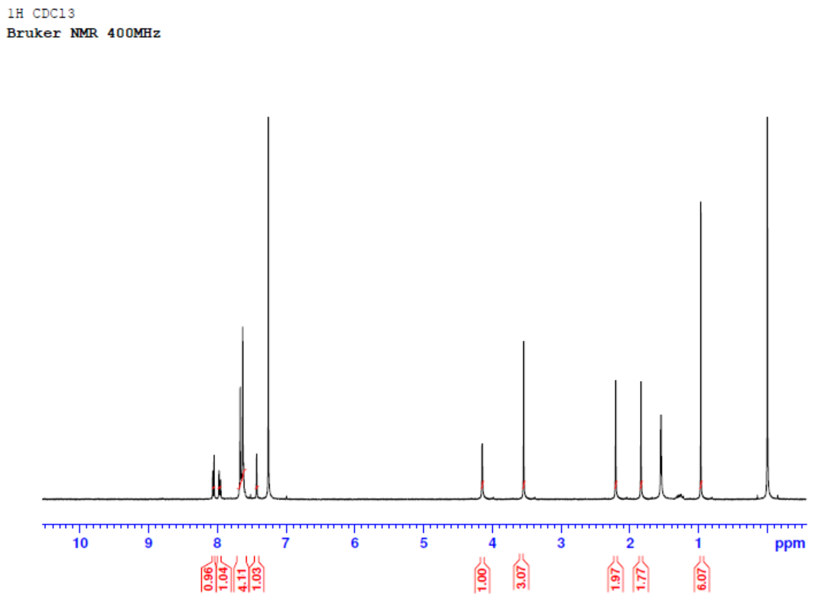

^{1}H NMR spectrum of compound **4a**

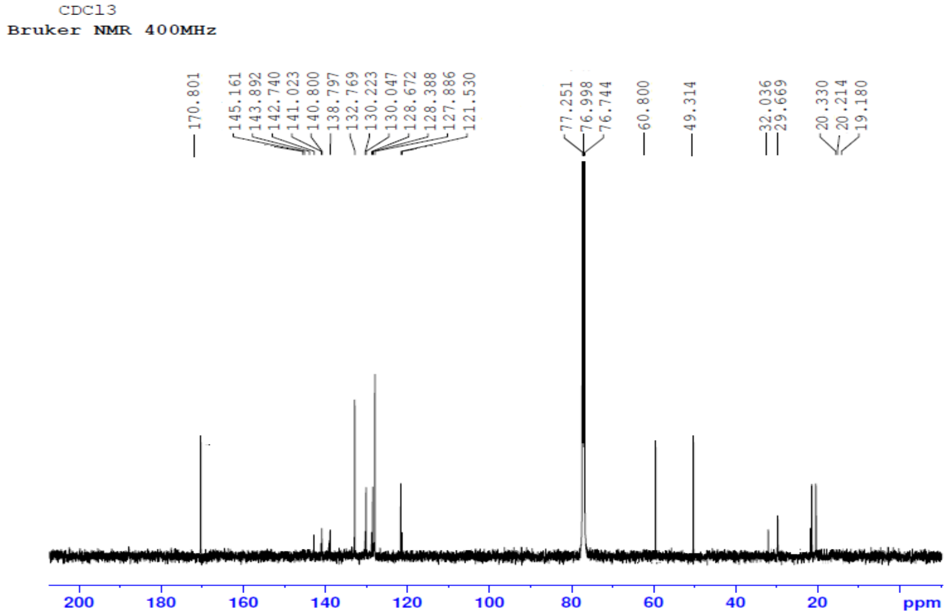

^{13}C NMR spectrum of compound **4a**

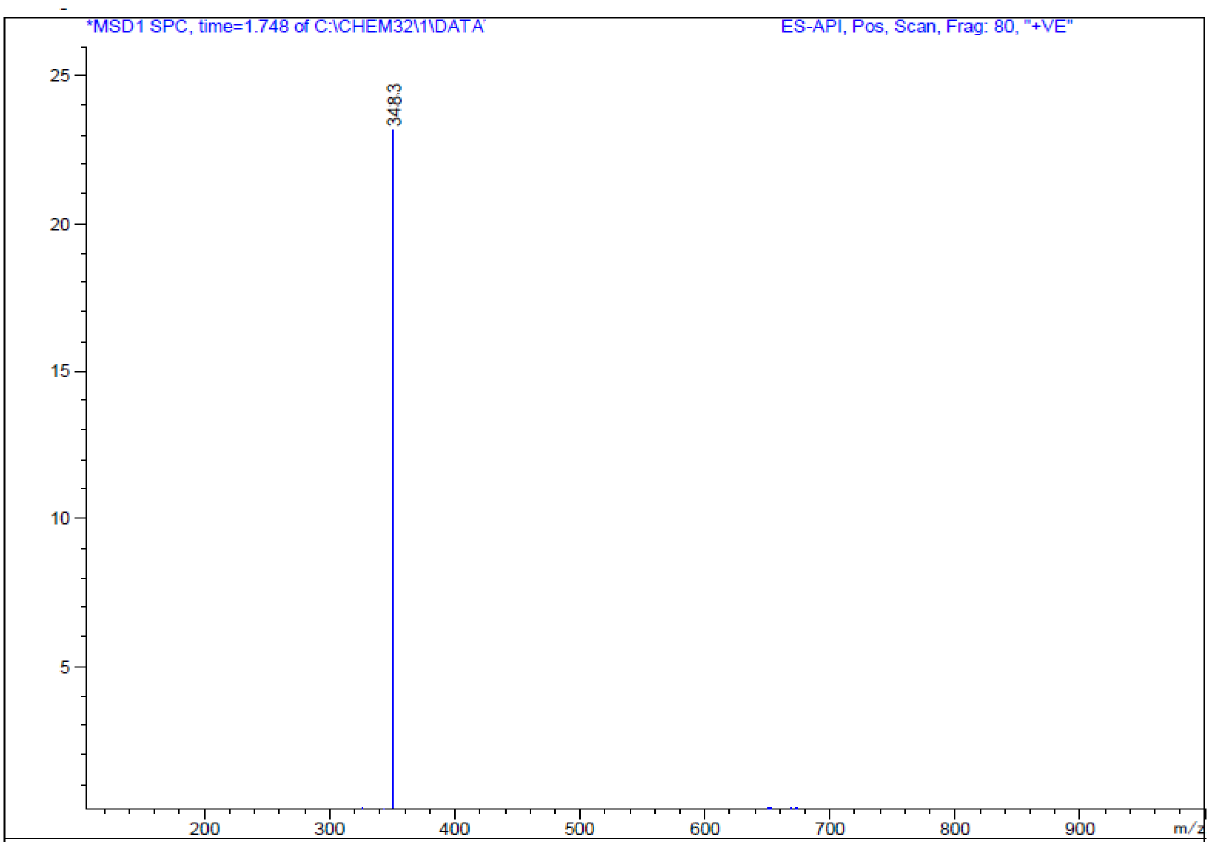

Mass spectrum of compound **4a**

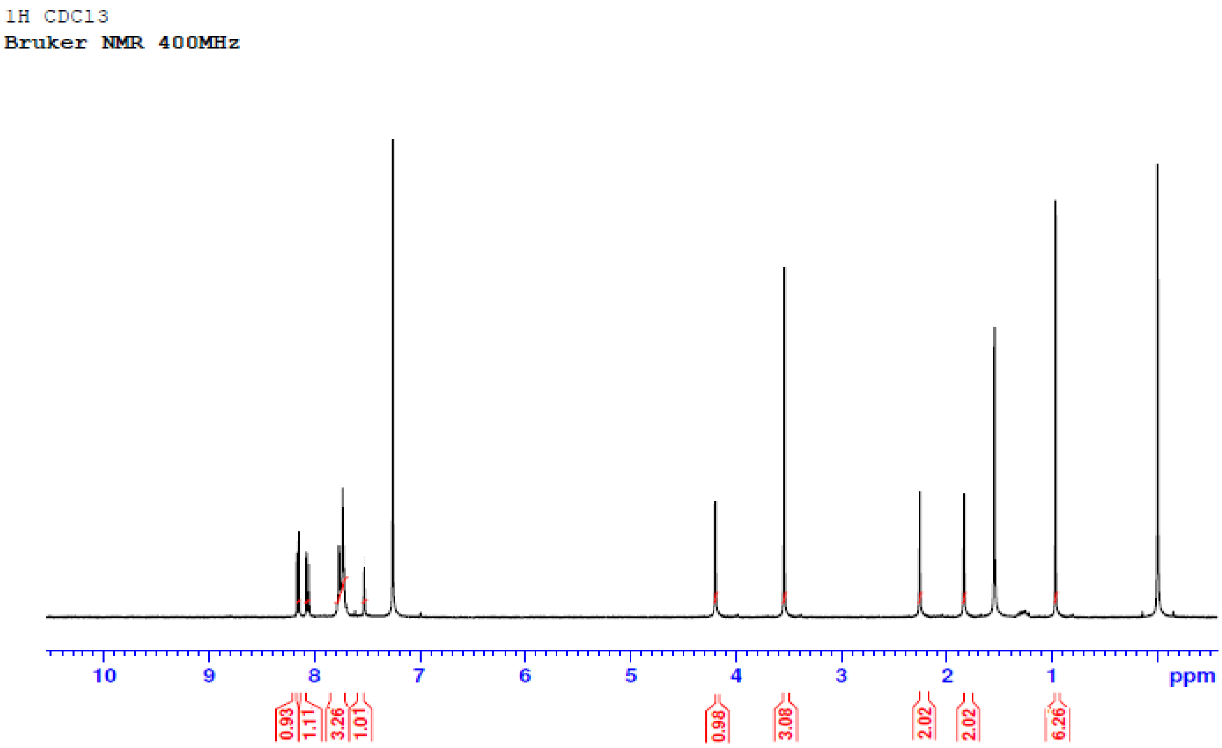

^{1}H NMR spectrum of compound **4c**

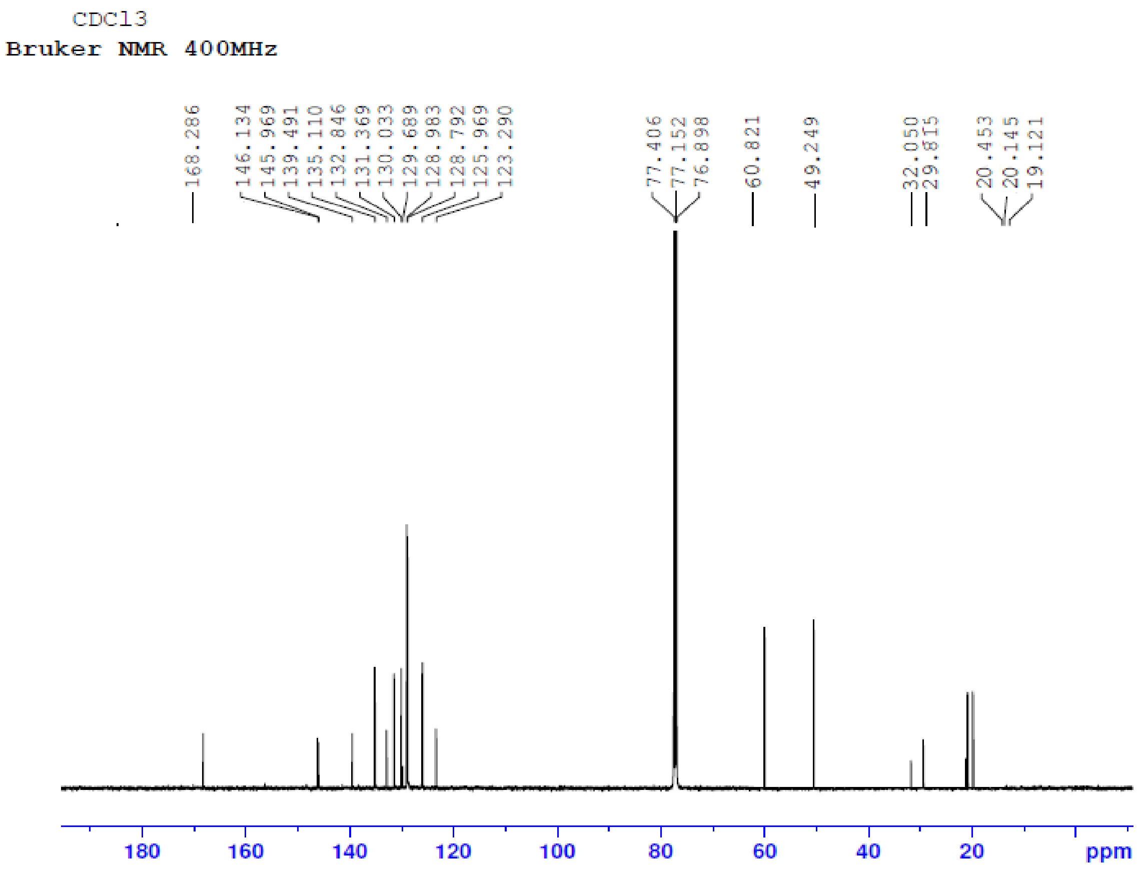

^{13}C NMR spectrum of compound **4c**

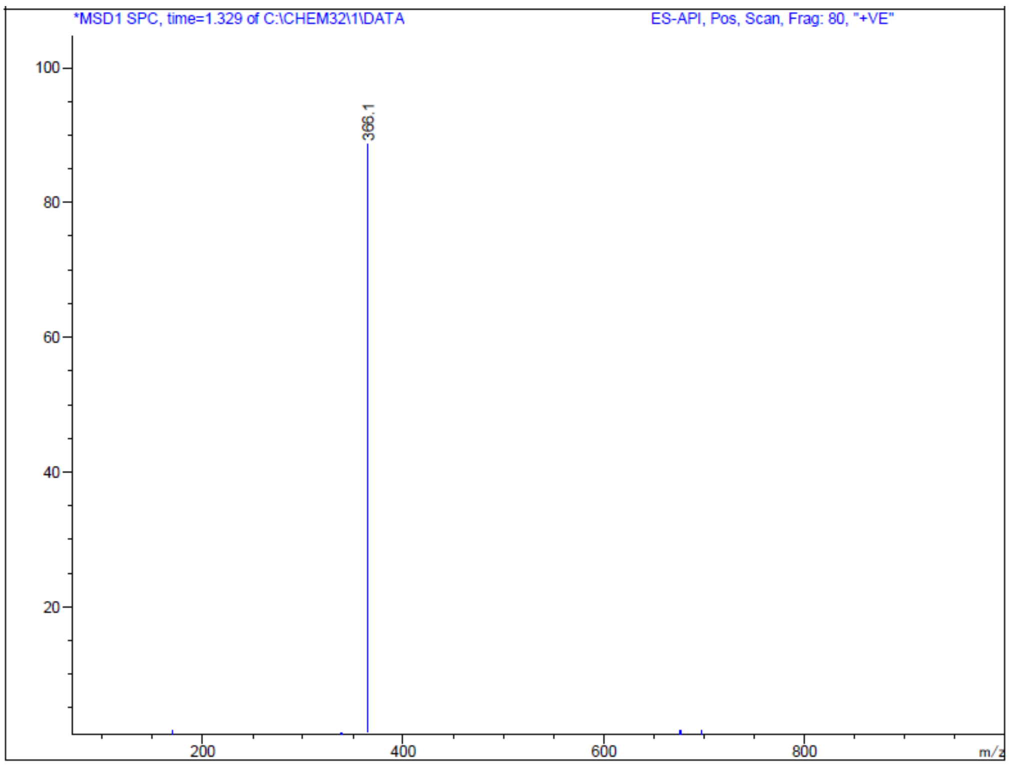

Mass spectrum of compound **4c**

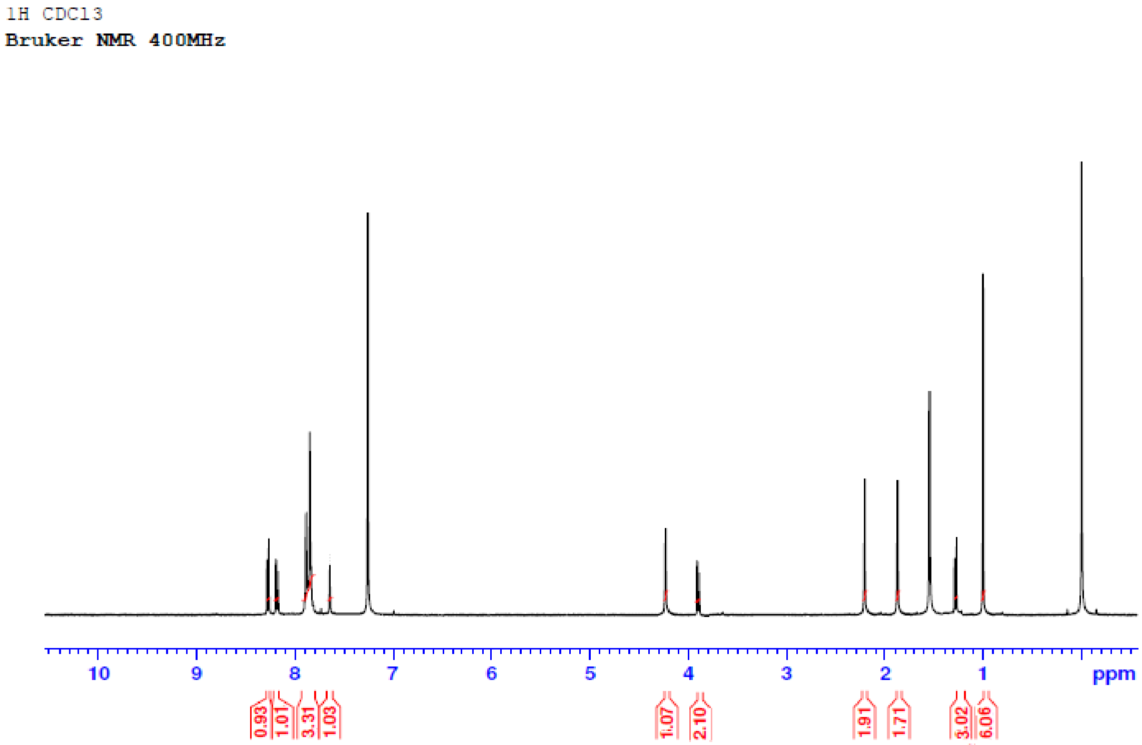

^{1}H NMR spectrum of compound **4d**

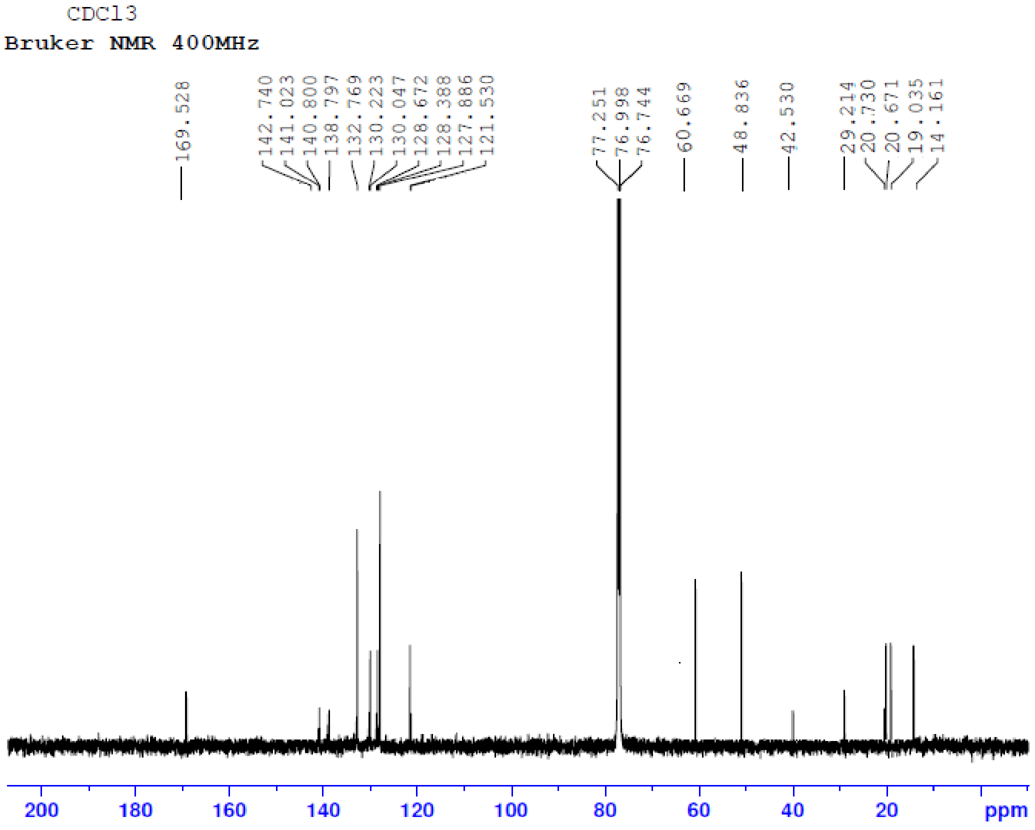

^{13}C NMR spectrum of compound **4d**

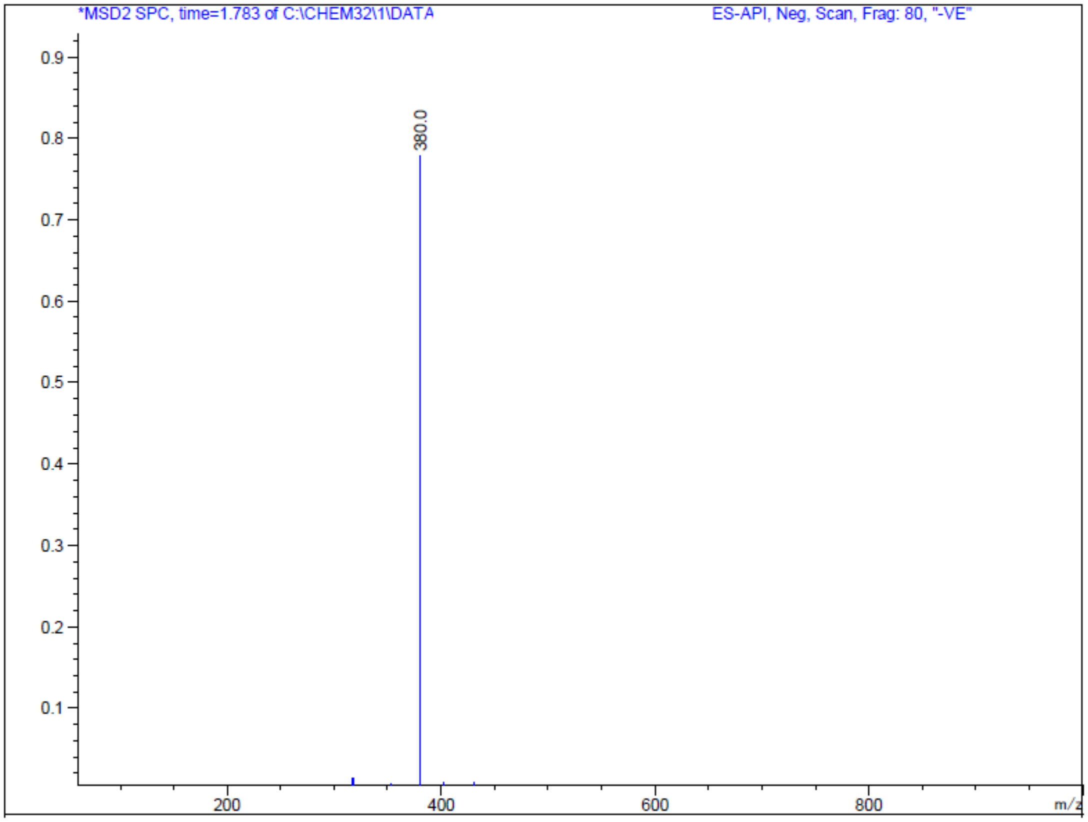

Mass spectrum of compound **4d**

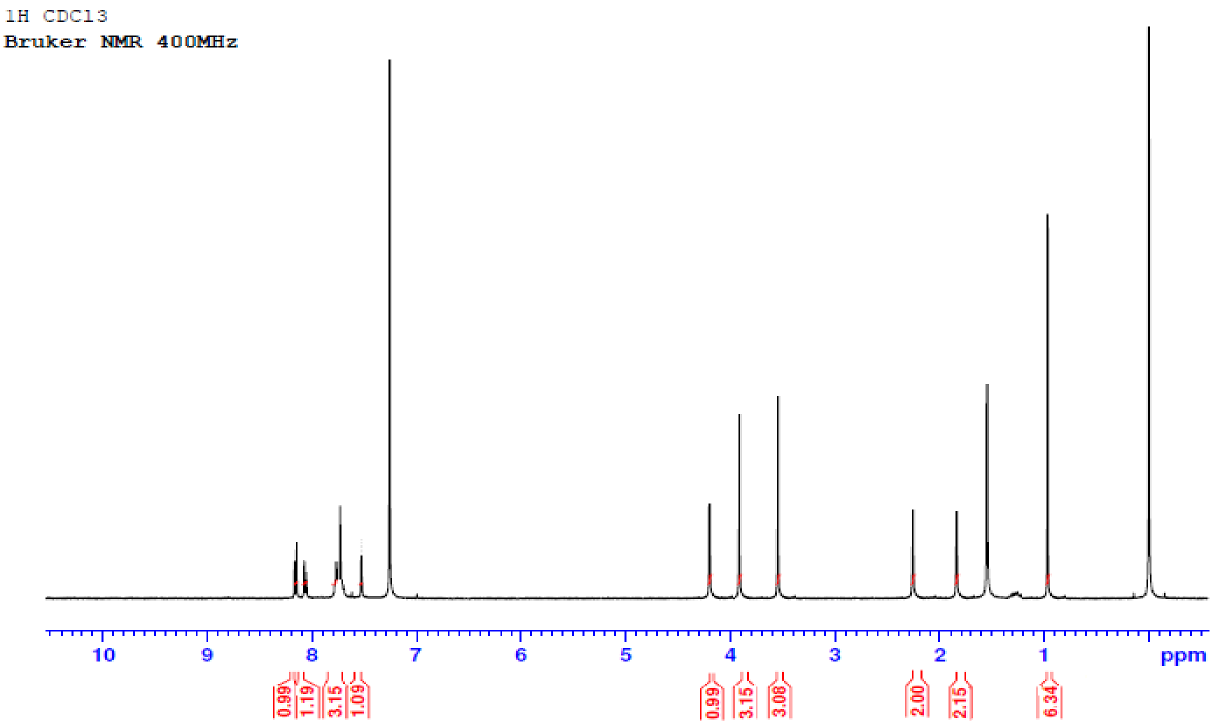

^{1}H NMR spectrum of compound **4g**

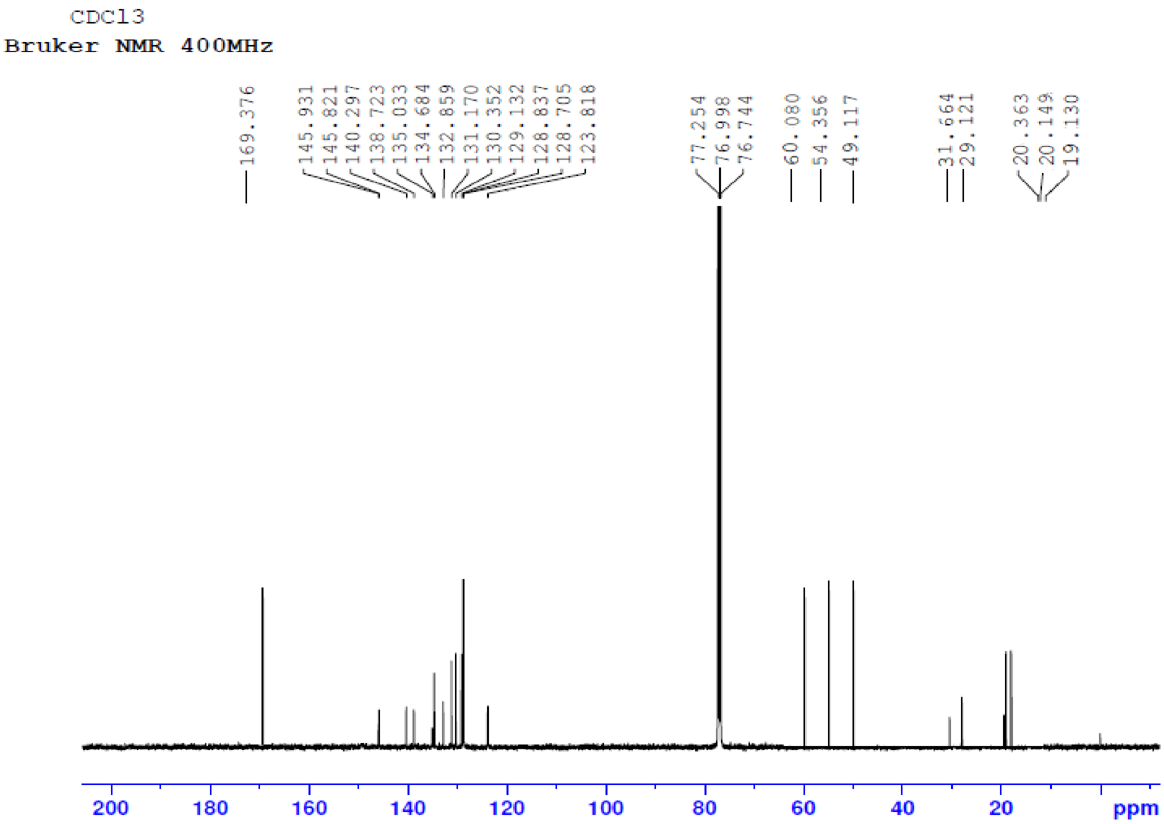

^{13}C NMR spectrum of compound **4g**

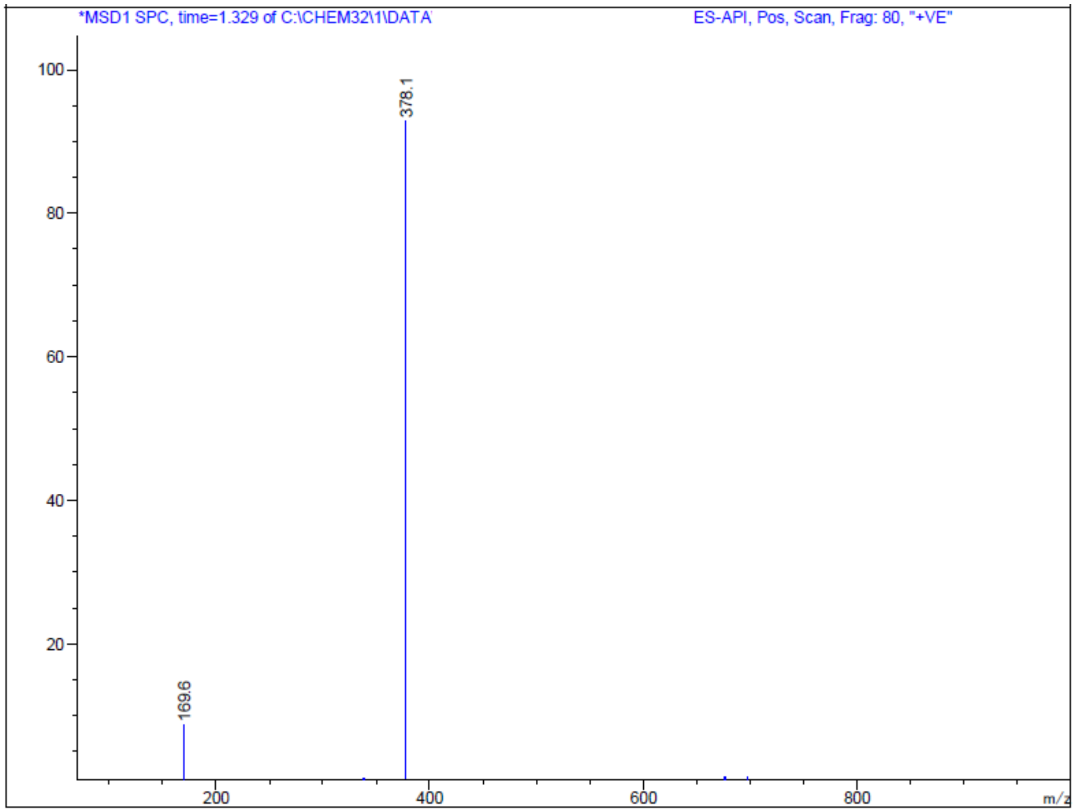

Mass spectrum of compound **4g**
